# Analysis of serum circulating MicroRNAs level in Malaysian patients with gestational diabetes mellitus

**DOI:** 10.1038/s41598-022-23816-3

**Published:** 2022-11-24

**Authors:** Sajad Jamalpour, Shamsul Mohd Zain, Reza Vazifehmand, Zahurin Mohamed, Yuh Fen Pung, Hesam Kamyab, Siti Zawiah Omar

**Affiliations:** 1 The Pharmacogenomics Laboratory, Department of Pharmacology, Universiti Malaya, Kuala Lumpur, Malaysia; 2 Department of Bioscience, Faculty of Medicine and Health Sciences, Universiti Putra Malaysia, Kuala Lumpur, Malaysia; 3 Department of Obstetrics & Gynaecology, University of Malaya, Kuala Lumpur, Malaysia; 4 Division of Biomedical Science, School of Pharmacy, University of Nottingham Malaysia, Selangor, Malaysia; 5 Malaysia-Japan International Institute of Technology Universiti Teknologi Malaysia, Jalan Sultan Yahya Petra, 54100 Kuala Lumpur, Malaysia

**Keywords:** Biochemistry, Biotechnology, Chemical biology, Genetics, Molecular biology, Diseases, Endocrinology, Molecular medicine

## Abstract

Gestational diabetes mellitus (GDM) is a severe global issue that requires immediate attention. MicroRNA expression abnormalities are possibly disease-specific and may contribute to GDM pathological processes. To date, there is limited data on miRNA profiling in GDM, especially that involves a longitudinal study. Here, we performed miRNA expression profiling in the entire duration of pregnancy (during pregnancy until parturition and postpartum) using a miRNA- polymerase chain reaction array (miRNA-PCRArray) and *in-silico* analysis to identify unique miRNAs expression and their anticipated target genes in Malay maternal serum. MiRNA expression levels and their unique potential as biomarkers were explored in this work. In GDM patients, the expression levels of *hsa-miR-193a*, *hsa-miR-21, hsa-miR-23a*, and *hsa-miR-361* were significantly increased, but *miR-130a* was significantly downregulated. The area under the curve (AUC) and receiver operating characteristic (ROC) curve study demonstrated that *hsa-miR-193a* (AUC = 0.89060 ± 04,470, *P* = 0.0001), *hsa-miR-21* (AUC = 0.89500 ± 04,411, *P* = 0.0001), *and miR-130a* (AUC = 0.6939 ± 0.05845, *P* = 0.0025) had potential biomarker features in GDM. *In-silico* analysis also revealed that *KLF* (Kruppel-Like family of transcription factor), *ZNF25* (Zinc finger protein 25), *AFF4* (ALF transcription elongation factor 4), *C1orf143* (long intergenic non-protein coding RNA 2869), *SRSF2* (serine and arginine rich splicing factor 2), and *ZNF655* (Zinc finger protein 655) were prominent genes targeted by the common nodes of miR23a, miR130, miR193a, miR21, and miR361.Our findings suggest that circulating microRNAs in the first trimester has the potential for GDM screening in the Malay population.

## Introduction

Gestational diabetes mellitus (GDM) is defined as any degree of glucose intolerance discovered during pregnancy^1^. Furthermore, GDM is a risk factor for maternal and fetal morbidity during pregnancy^1^. Babies born to women with GDM are more likely to have an excessive birth weight, known as macrosomia (weighing more than 4 kg), which can lead to juvenile obesity, type 2 diabetes, and/or cardiovascular disease later in life^2,3^. Globally, 21.3 million pregnancies are associated with hyperglycemia, with 18.4 million pregnancies related to GDM^4^. It has been shown that GDM is a complex disease with multiple etiologies^5^. Studies have shown that the development of GDM may be the result of a combined genetic and environmental effect, but the exact cause is still unknown^5^. GDM is diagnosed at the end of the second or early third trimester, depending on the physiological findings, and the generally accepted time for screening is the end of the second trimester, that is, between 24 and 28 weeks of pregnancy^6^. Over the past few decades, several studies have shown that aberrant expression of miRNAs is associated with pregnancy complications and the progression of GDM^7^, but longitudinal data on miRNA profiling in GDM is scarce. MiRNAs also function in blood glucose homeostasis and insulin production and secretion^8^. Furthermore, miRNAs have been reported to be present in biological fluids such as plasma/serum of diabetic patients and are highly stable there which can be easily detected and measured^9^. MicroRNA are short, single stranded RNA that post-transcriptionally regulate gene expression^10^. miRNAs produced in the nucleus are pri-miRNA and they are processed into pre-miRNA that is exported to the cytoplasm by exportin-5 and converted to a mature miRNA by Dicer complex and exert their action by targeting 3′ untranslated region (3′-UTR) of mRNA resulting in inhibition of protein synthesis^11^. Although GDM is diagnosed at the end of the second or beginning of the third trimester, using this diagnostic threshold, there is no opportunity to prevent pathological changes (accumulated damage) that may occur during first and second trimester (Undiagnosed Period). Furthermore, the implementation of screening tests such as microRNAs evaluation during early pregnancy affords opportunity to identify women at risk of disease and to evaluate intervention strategies on pregnancy outcome and the long-term health of both mother and baby^3^. In a systematic comprehensive review that was conducted in Malaysia, emphasised the high prevalence of GDM and recommended universal screening of this important health condition in pregnancy^12^. Furthermore, the diagnosis of GDM, which can be detected later in pregnancy and can result in major difficulties for both the mother and the fetus, is crucial in the early gestational weeks. This study aimed to evaluate changes in the expression of circulating miRNAs in the serum of patients with GDM during the first, second and third trimesters of pregnancy and subsequent changes in miRNA expression at postpartum period. To date, miRNA profiling from early diagnosis of pregnancy until parturition and postpartum has yet to be performed in GDM study.

## Results

### Participant characteristics

The results demonstrated that, the demographics factors such as weight, BMI.2-h Glucose and family history have a significant association with GDM in the current research. (*P* < 0.05). The demographics and clinical data of the 48 subjects (24 patients vs 24 controls) are shown in Table 1.

**Table 1:** Demographics and clinical data of the study population. Data presented as mean ± SD and differences in demographical data were evaluated using ANOVA and T- test. *P* < 0.05 = significant.

Characteristics	GDM (24)	Control (24)	*P*-Value
Maternal age/Years	31.42 ± 4.836	30.58 ± 3.844	0.4971
Weight	62.92 ± 14	49.36 ± 8.047	0.0005
BMI (kg/m2)	27.63 ± 4.388	21.11 ± 3.086	0.0001
FPG	5.375 ± 2.144	4.208 ± 0.2701	0.011
2-h glucose mmol/l	7.5 (6–8.5)	6.0 (5.1–6.4)	< 0.0001
Family history (diabetes)	20 (maternal)	3	0.0001
Birth weight/kg	3.270 (3100–3/5)	3.370 (3.150–3.700)	0.167

### MiRNA expression profiles in GDM patients

**Figure 1:**
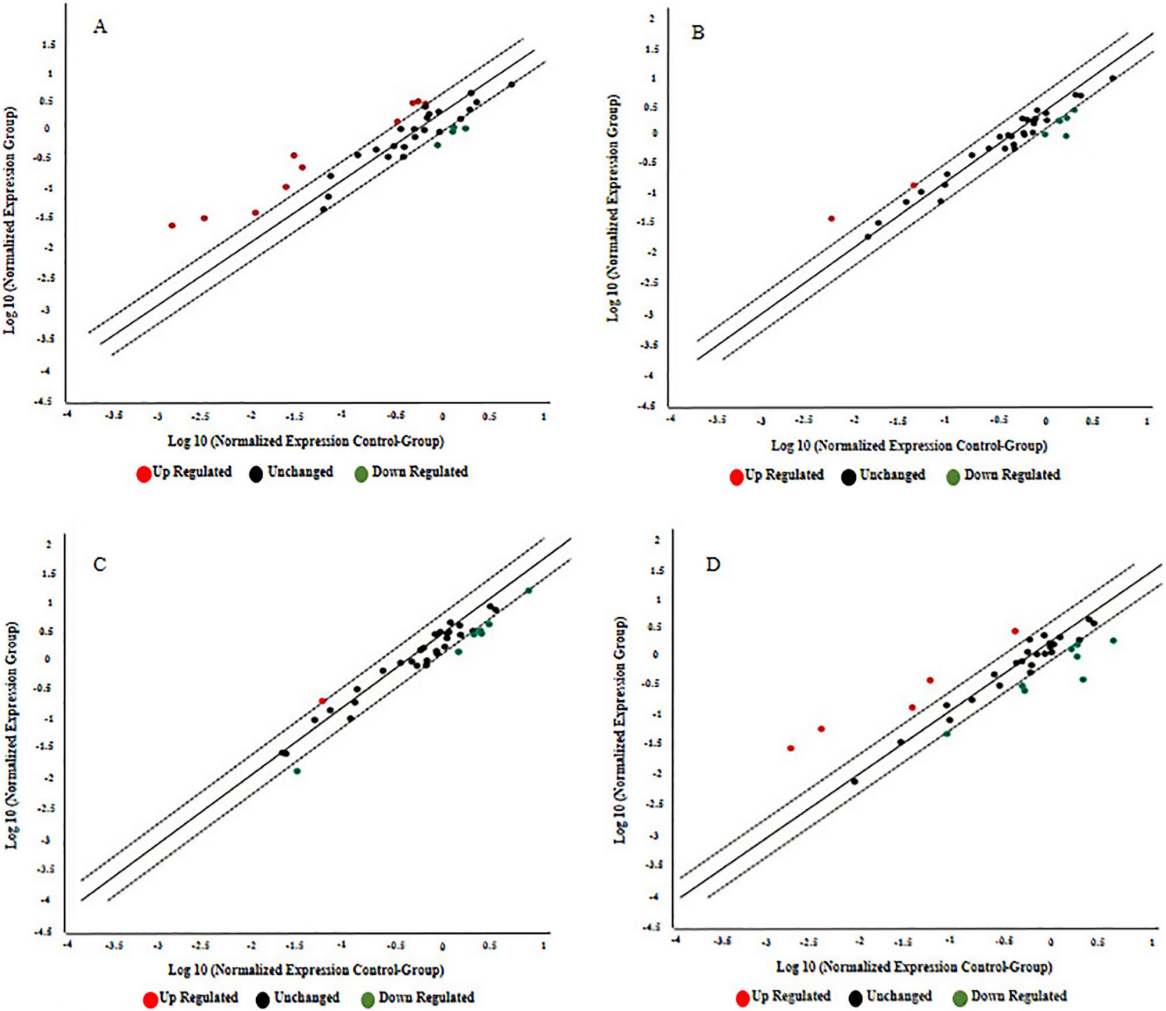
Scatter plot analysis of the relative expression of thirty-seven miRNAs compared between control and GDM patients. (**A**) = first trimester, (**B**) = second trimester (**C**) = third trimester (**D**) = post-partum. Color scheme: red color indicates upregulated miRNAs, while blue indicates downregulated miRNAs. The miRNAs that are significantly altered between the two groups (control and GDM) are located above. The diagonal black line corresponds to *P* < 0.05. Analysis was performed using online statistical analysis by Qiagen.

**Figure 2:**
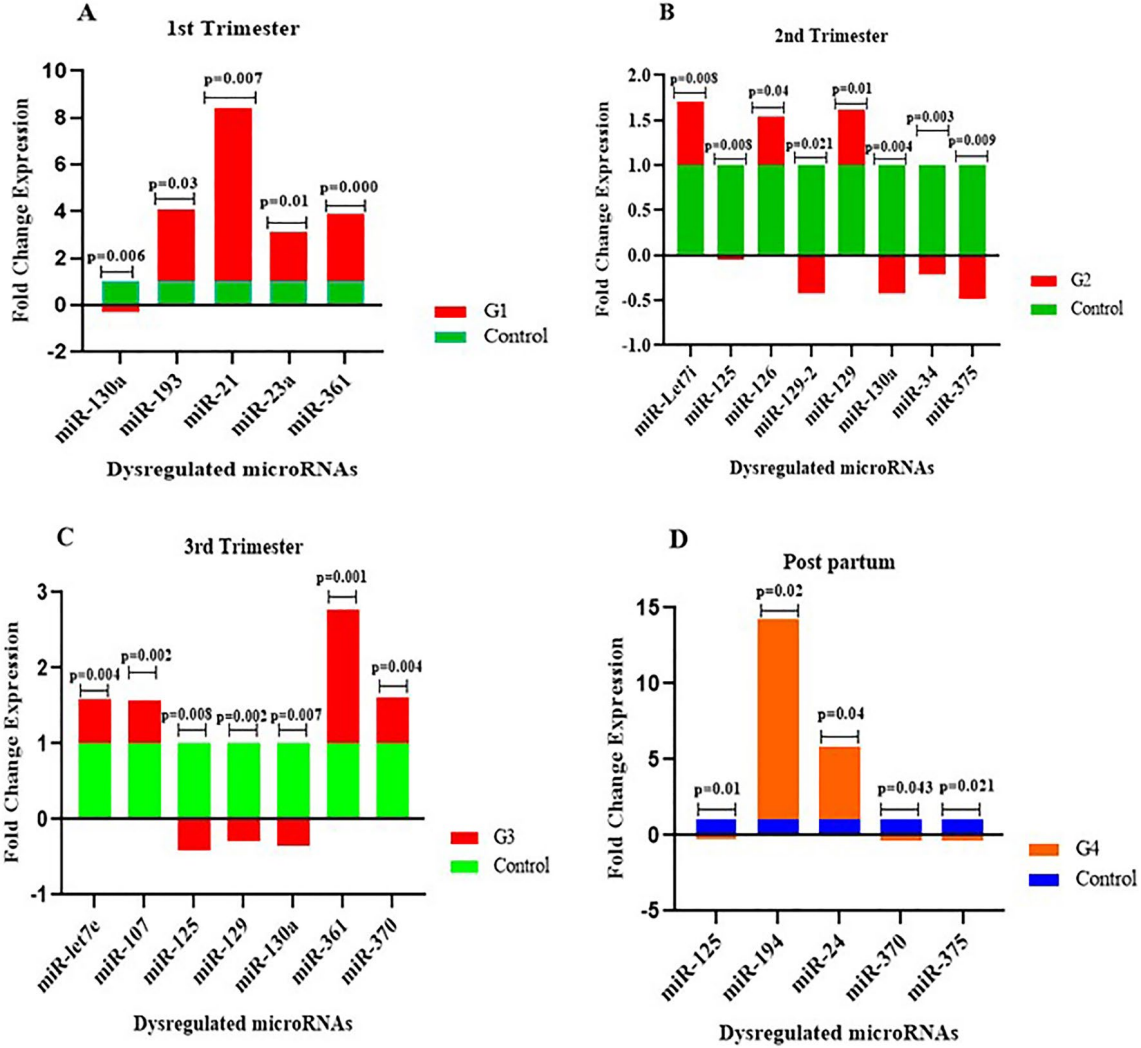
Dysregulated expression pattern of microRNAs in First (**A**), Second (**B**), Third (**C**) and postpartum (**D**) in GDM patients. Charts were derived from Graf Pad Prism version8. *P*-value calculated using non-parametric analytical test.

The results of the miScript miRNA PCR Array revealed a significant dysregulation pattern of miRNAs in the first, second, third and postpartum periods (results presented in Table 2). In the first trimester, four miRNAs, namely *hsa-miR-193a*, *hsa-miR-21, hsa-miR-23a*, and *hsa-miR-361*, were significantly upregulated while only *has-miR-130a* was significantly downregulated in GDM patients. In the second trimester, *hsa-miR-let7-i*, *hsa-miR-126, hsa-miR-129, were* significantly upregulated and *hsa-miR-125, hsa-miR-129–2, hsa-miR-130a, hsa-miR-34 and hsa-miR-375* were significantly downregulated*.* In the third trimester*,* upregulation was observed with *hsa-miR-let7e, hsa-miR-107, hsa-miR-361* and *hsa-miR-370* while downregulated miRNAs were *hsa-miR-125, hsa-miR-129* and *hsa-miR-130a.* In the postpartum period, data analysis revealed that two microRNAs *hsa-miR-194* and *hsa-miR-24* are significantly upregulated and three miRNAs *hsa-miR-125, hsa-miR-370,* and *hsa-miR-375* are significantly downregulated*.* A scatter plot and expression diagram of relative fold change showed the dysregulation patterns of miRNAs in a separate phase of GDM and control groups (Figs. 1 and 2). Implementation of screening tests such as miRNAs evaluation during early pregnancy, offers an opportunity to identify women at risk of GDM and to evaluate intervention strategies on pregnancy outcome and the long-term health of both mother and baby. Therefore, we focused more on the results from the first trimester. Dysregulated microRNAs in the first trimester are presented in Table 3.

**Table 2:** Dysregulated microRNAs in each trimester and post-partum period of GDM.

Dysregulated miRNAs	Trimesters							
	1st		2nd		3rd		Post-partum	
	Fold change	*P*-Value	Fold change	*P*-Value	Fold change	*P*-Value	Fold change	*P*-Value
miR-130a	− 0.32	0.006	−0.43	0.004	−0.34	0.007		
miR-193a	3.1	0.03						
miR-21	7.4	0.007						
miR-23a	2.14	0.01						
miR-361	2.9	0.000			1.77	0.001		
miR-let7i			0.71	0.008				
miR-125			−0.047	0.008	−0.42	0.008	−0.21	0.01
miR-126			0.55	0.04				
miR-129-2			−0.42	0.021				
miR-129			0.62	0.01	−0.29	0.002		
miR-34			−0.22	0.003				
miR-375			−0.48	0.009			−0.31	0.021
miR-let7e					0.59	0.004		
miR-107					0.57	0.002		
miR-370					0.61	0.004	−0.33	0.043
miR-194							13.27	0.02
miR-24							4.85	0.04

**Table 3:** MiRNAs role and their putative and predictive target genes in the first trimester of GDM.

microRNAs symbol	Putative and predictive target genes/Target score98-100)	microRNA’s role in GDM	Current study	
			Expression pattern/FC	*P*-Value
*miR-130a*	*SRSF2, CLIP1, GJA1, CPEB1, SLAIN1, SKIDA1, ESR1, ACVR1, TSC1, RPS6KA5, MDM4, KLF, IGF1, RAP2C, ACSL4, PIK3CB, ZBTB20, MYBL1, DDX6, FAM155A, KBTBD8, ZBTB18, ZFYVE9,TBL1XR1,LGALSL, ATG16L1, RO60, CHST1, MECP2, FMR1, CNOT6, SOS2, DYNC1LI2, SECISBP2L,AMPKa,RunX3*	Inhibition of glucose uptake Impaired mitochondrial function and oxidative stress which affects fetal development^16^	Decreased/−3.12	0.006548
*miR-193a*	*SRSF2, MAPK10, PIGA, DCAF7, RAPGEF6, KLF, AFF4,EFNB2*	Promote trophoblast migration and invasion^39^	Increased/3.1	0.032241
*miR-21*	*C1orf143, ZNF25, YOD1, PRDM11, FASLG, ZNF367, VCL, SKP2, TGFBI,PPARα*	Down regulated of miR-21 Inhibit Cell Proliferation and Infiltration^40^, increased miR-21 is associated with pregnancy complications^41^	Increased/7.4	0.007121
*miR-23a*	*ZNF25, ZNF99, SEMA6D, FAM234B, TRIL, INTU, ZNF138, AUH, TAB3, SEC23IP, ZBTB34, PDE7A, PPARGC1A, PDE4B, TOP1, KLF, TMED5, FUT9, SESN3, DNAJC6, PPM1K, VGLL3, SFT2D1, ZNF716, C2orf69, ATP11C, NEK6, LPP, ARHGAP20, MRC1, ETNK1, WBP2, FAM126B, TMPO, PPP4R4, HEXIM1, CCNT2, PKP4, PTEN, TNRC6A, SLC4A4, MICU3, RAB8B, CACUL1, PPIF, ZNF117, REPS2, CSNK1G3, ZFHX4, SLC1A1, MAP4K4, ROBO2, PRTG, NUFIP2, RPRD2, CTCF, SCG5, SEC24A, CDC40, BORA, CNOT6L, NCOA2, NUP50, NACC2, ZNF655*	Potential biomarkers for GDM in the first trimester^25^	Increased/2.14	0.018739
*miR-361*	*PIK3CG, TFAP2B, ZMAT3, RANBP17, AFF4, ZNF655, C1orf143*	Ultimately is a sex specific early markers of extremely low gestational age^42^	Increased/2.9	0.000063

### ROC curve analysis

**Figure 3:**
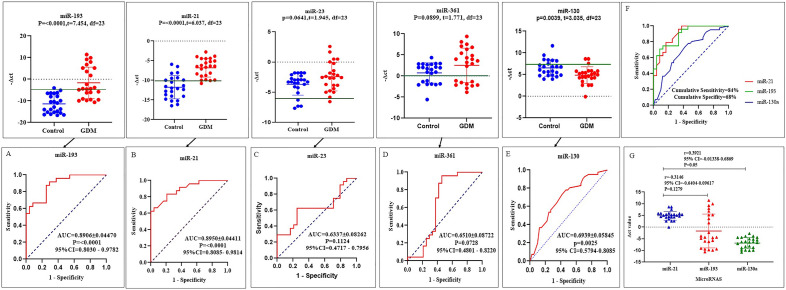
ROC curve analysis. The figure shows the comparison of five dysregulated microRNAs and their specific AUC based on their ΔCt distribution in the first trimester of pregnancy (**A**–**E**). Cumulative Graff of ROC analysis of three combined microRNAs and Binary logistic regression (BLR) analysis (**E** and **F**). The ROC curve is derived from Graf Pad Prism version 8. Normality distribution of samples was performed using Shapiro–Wilk test. *P*-value calculated using non-parametric analytical test.

The diagnostic evaluation of ROC curve based on distribution of ΔCt of dysregulated microRNAs in the 1st trimester in GDM patients revealed that the area under the curve (AUC) for *hsa-miR-193a, hsa-miR-21, hsa-miR-23a*, *hsa-miR-361*and *hsa-miR-130*a were 0.8906 ± 0.04470 (*P* < 0.0001, 95% CI = 0.8–0.9), 0.8950 ± 0.04411(*P* < 0.0001, 95% CI = 0.8–0.9),0.6337 ± 0.08262 (*P* = 0.1124, 95% CI = 0.4–0.7), 0.6510 ± 0.08722(*P* = 0.07, 95% CI = 0.4–0.8) and 0.7222 ± 0.07450 (*P* = 0.0083, 95% CI = 0.5–0.8) respectively. The discriminatory capability (*P* < 0.05) of miR-193a, miR-21 and miR-130a had the highest area under the curve (AUC) (Fig. 3A–E). The data indicated that three microRNAs namely, hsa-miR-193a (Sensitivity of 91% and Specificity of 70%, *P* = 0.0001), hsa-miR-21(Sensitivity of 83% and Specificity of 79%, *P* = 0.0001) and hsa-miR-130a (sensitivity of 79% and Specificity 54%, *P* = 0.0025) fit the biomarker role and have a potential diagnosis value for GDM detection in the first trimester of pregnancy. Further analysis revealed a decreasing AUC to 0.84 ± 0.021 when were incorporated into a 3-miRNA model (hsa-miR-193a, hsa-miR-21, and hsa-miR-130a signature (Fig. 3F). Furthermore, based on regression analysis, a significant correlation had been revealed between miR-21 and miR-130a (r = 0.3921, *P* = 0.05) whereas here was not observed significant association between mi-R21 and miR-193a (r = 0.3146, *P* = 0.1279) (Fig. 3G).

### *In-silico* target analysis

**Figure 4:**
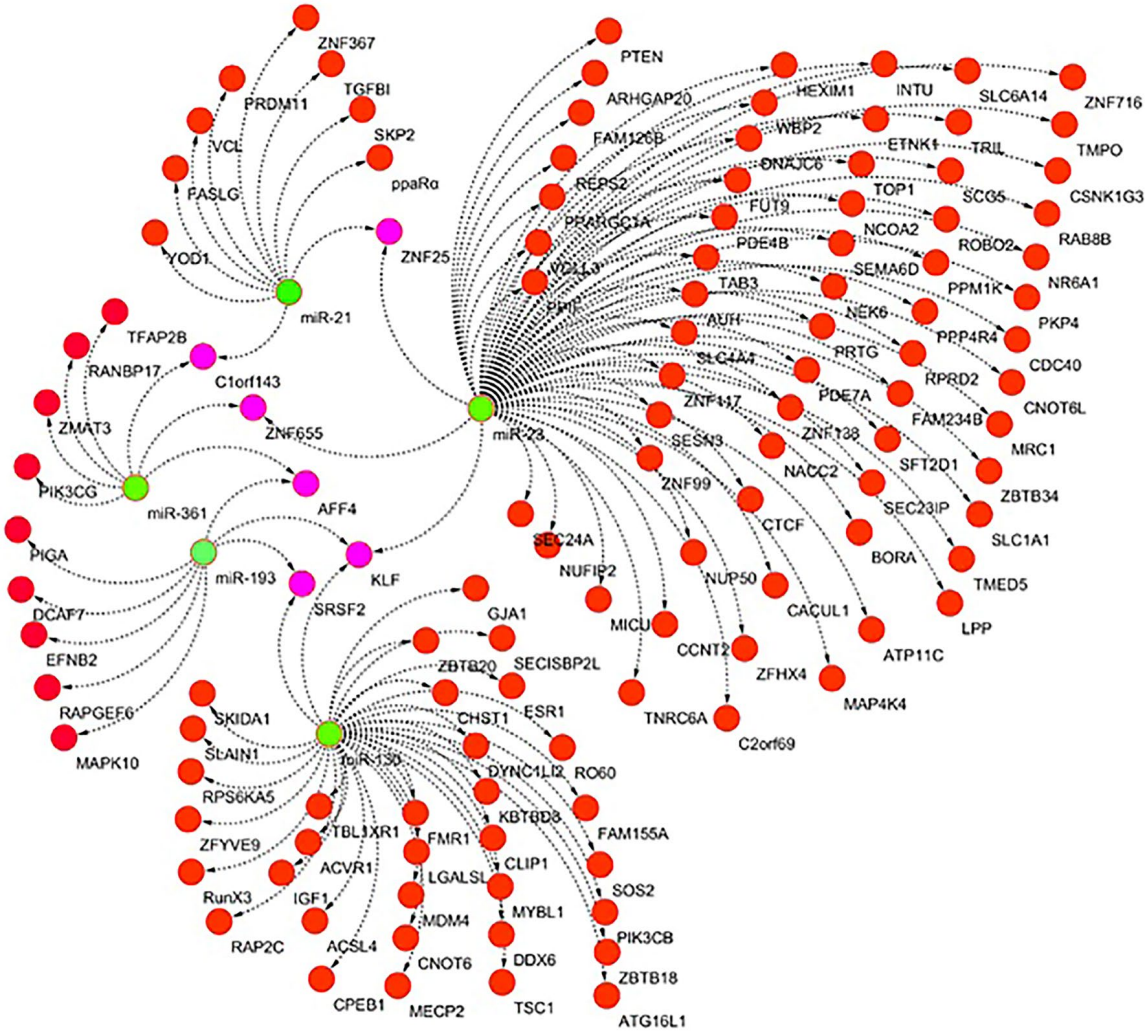
*In-silico* analysis of dysregulated microRNAs in the first trimester. Green and purple colors indicate dysregulated microRNAs and their common target genes, respectively. *ZNF25, RANBP17, CLORF43, ZNF655, AFF4, KLF* and *SRSF2* were targeted in a common node of microRNAs. Interaction pathway provided by Cytoscape tool version 3.7.1.

In silico analysis revealed the interaction between miRNAs and their target genes involved in the first trimester of GDM. In this analysis, *KLF, ZNF25, AFF4, SRSF2*, C1orf143 and *ZNF655* were prominent genes targeted by the common nodes *miR23, miR130a, miR193, miR21*, and *miR361* (Fig. 4).

## Discussion

GDM is an increasingly common condition and can cause serious and lifelong harmful complications for both mother and child. There is great interest in the potential role of miRNAs as regulators of biological processes, mediators of tissue crosstalk, and biomarkers of GDM^12^. MiRNAs participate in various mechanisms associated with pregnancy and GDM. In addition, in maternal blood, circulating miRNAs are persistent and detectable. As a result, they are potential biomarker candidates for non-invasive pregnancy problems diagnostic tests. Studies have shown that dysregulated miRNAs are associated with pregnancy complications, suggesting the potential use as prognostic markers for GDM disease^13,14^. Furthermore, previous studies have reported that serum or plasma miRNAs are differentially expressed between GDM patients and controls.

In this study, we attempted to identify the differentially expressed serum-derived miRNAs and the molecular interactions of the *in-silico* miR target genes that may be involved in GDM. Furthermore, the potential characteristics of dysregulated miRNAs were investigated. GDM is diagnosed at the end of the second or beginning of the third trimester^3^, leaving no time to prevent pathological changes that could occur during the first and second trimester. Thus, miRNAs detection as early as in the first trimester is crucial and was our main study objective. Our results showed that miR130a is significantly downregulated in the GDM sample, while miR193a, miR21, miR23a and miR361 were significantly upregulated in the first trimester.

One of the prominent and thought-provoking results of the current research is the biological behaviour of miR-130a, which has decreased expression in the first, second, and third trimesters of pregnancy while there were no changes in expression level in the postpartum period (Fig. 2). MiR130a affects a variety of cellular processes, from inhibition of glucose uptake, mitochondrial function, oxidative stress to fetal development^15,16^. In general, this microRNA has a dual role in biological processes. Due to its cellular functions, miR-130a expression is dysregulated in a variety of pathologies, including oral squamous cell carcinoma^17^, ovarian epithelial cell carcinoma, thyroid eye disease^18^, cervical cancer^19^, and acute myeloid leukemia^20^. It was also shown that upregulated miR-130a reduces intracellular ATP levels in the pancreatic beta cell^21^. Furthermore, previous studies found upregulation of circulating miR-130a has a crucial role in diabetes-related complications^22^. In a study by Meng et al., decreased miR-130a in endothelial progenitor cells from diabetes mellitus was reported to contribute to impaired EPC (Endothelial progenitor cell dysfunction) function via its target RunX3^23^.

MiR23a identified from this study, has been proposed as a potential biomarker for early diagnosis of prediabetes and type 2 diabetes and is particularly useful in distinguishing between undiagnosed and prediabetes^24^. In a research study that conducted by Liron Yoffe et al^25^, miR-23a was in upregulated pattern that the ROC value (AUC = 0.89 and accuracy = 0.90) revealed its biomarker characteristics in Spain and Italy countries patients with GDM (Liron Yoffe et al. 2019). Furthermore, our finding was in an agreement with Yang et al. in which we found that miR-23a could function as a potential biomarker in the first trimester of GDM. On the other hand, upregulated miR193a has been shown to play a vital role in the pathogenesis of placenta accreta spectrum development mediated by the target in *EFNB2* gene via the EMT signaling pathway^26^. In addition to that, elevated miR193a was reported as a potentially new biomarker for the diagnosis of diabetic nephropathy^27^. Although miR193a was upregulated in the current study, and its biomarker potential was confirmed in other disease conditions, but its etiologic role in GDM is unknown and requires further study.

Literatures proven that miR21 has a significant biological route in a variety of diseases, although, as a molecular diagnostic marker can therapeutically regulate type 2 diabetes and pancreatic cancer^28^. However, it is still unclear how miR21 is linked to GDM. One plausible explanation could be that miR21 promotes glucose uptake through induction of the PPARα gene in GDM patients and inhibits cell proliferation and infiltration^29^. Gestational-adjusted expression of *miR-21* has been reported to be positively associated with GDM^30^. Sexually dimorphic miRNA expression during pregnancy in the human placenta was reported by Amy E. Flowers et al. They found that miR361 was differentially expressed and upregulated in women of the 1st and 3rd trimesters^31^. One of the significant points in this research is the results of the binary logistic regression and correlation analysis. BLR shows that the accumulation of the effect of three microRNAs causes a significant decrease in their biomarker power, which can be due to the difference in the expression value of microRNA genes in our study population. In addition, the results of the correlation analysis among three microRNAs signature showed a significant relationship between the expression of miR-21 and miR-130a only, so that the increase of miR-21 is associated with the decrease of miR-130a in GDM patients in the first trimesters of pregnancy, and based on our knowledge, these findings are for the first report in the Malay population.

Overall, our study data investigated differentially expressed microRNAs in GDM patients and we showed the potential role of *hsa-miR-193a, hsa-miR-21*and *hsa-miR-130a* as biomarker but the role of these dysregulated microRNAs in the pathogenesis of GDM is still not clearly understood.

This research has several limitations. Since miRNA profiling experiments are relatively expensive, they are frequently conducted on a small number of research participants. Furthermore, there are very few miRNAs profiling studies in GDM, especially for longitudinal research. Only four published reports on miRNA profiling in GDM were discovered during our initial search. However, three^32–34^ were only done during the first trimester, while a fourth^30^ was done throughout both the first and second trimesters. Data on miRNA expression in different trimesters are therefore hard to come by. This work is limited by the absence of follow-up on the functional aspects of the miRNA findings. In addition, we did not compare the circulating miRNAs with the placental miRNAs. This is due to the religious belief that disapproved tissue sampling. Future research must investigate the functional role of these miRNAs and their correlation with GDM parameters. In addition, it is essential to investigate the placenta miRNAs for comparative study.

The routine use of miRNAs as screening tools has enormous promise for assisting in the earlier detection and treatment of GDM by dietary changes or pharmacological intervention. Their clinical usefulness in different diseases has been shown in an increasing number of research. MiRNA profiling during GDM is currently inconclusive, partly because of the scarcity of data and the lack of consistency among research' findings. Before circulating miRNAs are used in therapeutic settings, there are numerous analytical and pre-analytical hurdles that must be overcome. Pre- and post-analytical procedures should be standardized to improve reproducibility between experiments. Large prospective cohort studies should also be conducted to examine the factors influencing miRNA expression and determine whether they may be candidates for diagnostic or prognostic information.

## Conclusions

In summary, this study identified three miRNAs that are differently expressed in GDM women and potentially serve as biomarkers. It is hypothesized that these microRNAs regulate genes including *ZNF25, KLF* and *SRSF2.* Our findings emphasize the significance of these microRNAs, in particular their role in the first trimester of pregnancy, where such knowledge could be valuable for the early detection of GDM. However, the size of samples in the detection of biomarker potential of microRNAs was one of the limitations; the functional involvement of these miRNAs in the pathophysiology of GDM requires additional investigation. A comparative investigation with placenta miRNAs would improve the findings even further.

## Methods

### Participant characteristics

A case–control study was conducted on a total of 1122 women (267 GDMs and 855 controls) who had spontaneous deliveries at the University of Malaya Medical Center (UMMC) from April 2014 to June 2016. All participants were characterized by pregnancy, were nonsmokers, and did not abuse alcohol. The selection criteria for the study were the age of the mother between the ages of 18 and 45 and the diagnosis of gestational diabetes by a trained doctor. The age of mothers between the ages of 18 and 45 with normal pregnancies served as a control. Abnormal fetal, still giving birth, sickle cell anemia, thalassemia or other hemoglobinosis, and other pregnancies such as lupus, hypertension, thyroid disease, cardiovascular disease, transplantation, kidney disease, asthma or other serious disease Women diagnosed with previous conditions, drug abuse, a history of smoking and depression, and carriers of blood-borne infections were excluded. Screening was performed between the 24th and 28th weeks of gestation using the Modified Oral Glucose Tolerance Test (mOGTT). Universal screening includes pregnant women with a BMI greater than 27 kg/m^2^, previous giants weighing 4 kg or more, previous GDM, one-time relatives with diabetes, a history of unexpected prenatal fetal death, and birth defects. It was performed on pregnant women with a medical history Positive. GDM is defined as fasting plasma glucose (FPG) (≥ 5.1) and 75 g mOGTT plasma glucose (≥ 7.8). Substantial risk women with normal initial screening results were subjected to a repeat mOGTT at 4–6 weeks later. The GDM screening followed the National Obstetric Registry (NOR) guideline^35^. The control consisted of uncomplicated pregnant mothers who gave birth to a baby between 38 and 41 weeks. Trained personnel assessed the physical health of the mother and gestational age was calculated from the first day of the last menstrual cycle or from the patient's early ultrasound scan results. All study protocols followed the medical ethics from the Universiti Malaya Medical Centre (Ethics Committee/IRB Reference Number: 982.3), and informed consent was obtained from all participants.

### Serum RNAs extraction and cDNA synthesis

Twenty-four women with GDM were included from the pool randomly. Maternal samples were collected within first, second, third trimesters and 2–6 months postpartum. Twenty-four healthy pregnant women served as controls, taken randomly from the pool. The sample size was estimated based on publication by Kok et al^36^. Total RNAs were extracted from serum samples using the miRNeasy serum/plasma kit and synthesized into cDNA using the miScript II Rt kit according to the manufactures protocol (Qiagen, Mississauga, Ontario, Canada).

### MiRNAs expression profiling

A pathway-focused miScript miRNA PCR Array Human Diabetes (Qiagen, Mississauga, Ontario, Canada) was used in combination with miScript SYBR Green PCR kit (Qiagen) to profile miRNA expression in a 96-well plate using a Step One Plus™ real time PCR detection system (Applied Biosystem, California, USA) following the cycling conditions recommended by the manufacturer’s instructions. Amplification conditions were 15 min at 95 ℃, followed by 40 cycles of 15 s at 94 ℃, 30 s at 55 ℃, and 30 s at 70 ℃. A total of 37 miRNAs involved in GDM phenotype were selected for the PCR array. Six snoRNA/snRNA, including SNORD61, SNORD68, SNORD72, SNORD95, and SNORD96A were selected as housekeeping genes. Furthermore, miRTC and PPC genes served as reverse transcription control and primer assay positive control, respectively. PCR array reactions were conducted in triplicate repeats. The data were analyzed using online Gene Globe data analysis software version 2.1.0 (https://geneglobe.qiagen.com/). The validity and accuracy of array PCR were confirmed using reverse transcription-qPCR for five randomly selected miRNAs in triplicate.

### ROC curve analysis

A receiver operating characteristic (ROC) which is a plot of the true positive rate (Sensitivity) in function of the false positive rate (100-Specificity) for different cut-off points of a parameter was performed to evaluate the potential microRNAs biomarker characteristics.

### Insilico target genes Identification and bioinformatic analysis

Target Scan Human Release 8.0^37^ and miRDB ^38^were used to obtain predicted or previously experimentally validated miRNA target genes. Interaction network analysis was performed using the Cytoscape 3.7.1 tool to verify all potential interactions between the identified target gene and potential functional mediators.

## Data Availability

The datasets generated during and/or analyzed are available from the corresponding author on reasonable request.
