# Author Correction: Analysis of serum circulating MicroRNAs level in Malaysian patients with gestational diabetes mellitus

**DOI:** 10.1038/s41598-023-32260-w

**Published:** 2023-03-29

**Authors:** Sajad Jamalpour, Shamsul Mohd Zain, Reza Vazifehmand, Zahurin Mohamed, Yuh Fen Pung, Hesam Kamyab, Siti Zawiah Omar

**Affiliations:** 1 The Pharmacogenomics Laboratory, Department of Pharmacology, Universiti Malaya, Kuala Lumpur, Malaysia; 2 Department of Bioscience, Faculty of Medicine and Health Sciences, Universiti Putra Malaysia, Kuala Lumpur, Malaysia; 3 Department of Obstetrics & Gynaecology, University of Malaya, Kuala Lumpur, Malaysia; 4 Division of Biomedical Science, School of Pharmacy, University of Nottingham Malaysia, Selangor, Malaysia; 5 Malaysia-Japan International Institute of Technology Universiti Teknologi Malaysia, Jalan Sultan Yahya Petra, 54100 Kuala Lumpur, Malaysia

Correction to: *Scientific Reports* 10.1038/s41598-022-23816-3, published online 24 November 2022

The original version of this Article contained an error in the spelling of the author Sajad Jamalpour, which was incorrectly given as Sajad Jamal pour.

The original Article has been corrected.

